# Method for generating multiple risky barcodes of complex diseases using ant colony algorithm

**DOI:** 10.1186/s12976-017-0050-0

**Published:** 2017-02-01

**Authors:** Xiong Li, Wen Jiang

**Affiliations:** 1 School of Software, East China Jiaotong University, Nanchang, 330013 China; 2 Software School, Hunan Vocational College Of Science and Technology, Changsha, Hunan 410118 China; 3 College of Information Science and Engineering, Hunan University, Changsha, Hunan 410082 China

**Keywords:** Single nucleotide polymorphisms, Complex diseases, Ant colony algorithm, Epistasis

## Abstract

**Background:**

Susceptible barcode recognition plays an important role in the diagnosis and treatment of complex diseases. Numerous approaches have been proposed to identify risky barcodes involved in the progress of complex diseases. However, some methods only consider differences in barcode frequencies between the control and disease groups; as such, these methods may be partial or even wrong. For example, some barcodes with a high risk ratio yield a low frequency on cases or exhibit a high frequency on controls, which may unreasonable from a statistical point.

**Results:**

In our study, a stricter criteria, maximum discrepancy and maximum constituency, is designed to evaluate each barcode and ant colony algorithm is used to search combination space of epistasis. For complex diseases with multi-subtypes, our method can list several potential barcodes contributing to different subtypes of complex diseases. Another contribution of this work is to introduce a method for determining the length of barcodes and excluding noisy barcodes whose frequencies are abnormal. In addition, common pathogenic genes shared by different risky barcodes are also recognized, which may provide key clue for further study, such as gene function analysis.

**Conclusions:**

Experimental results reveal that our method can find multiple risky barcodes whose risk ratio and odds ratio are >1. These barcodes could be related to different subtypes of complex diseases.

## Background

Single nucleotide polymorphisms (SNPs) play an important role in the diversity of phenotypes [1]. Genome-wide association studies (GWAS) have been applied to reveal the effect of SNPs on complex diseases and to identify candidate susceptible genes, which are statistically correlated with specific complex diseases. After few candidate SNPs have been recognized through GWAS, risky SNP barcodes regarded as a genotype in multiple SNPs should be identified [2]. For case–control study with haplotype samples, the barcode is the same as haplotype in given SNPs. However, the barcode is also suitable for association study on genotype datasets. If an individual carries a disease-specific barcode, the individual can be regarded as a potential patient with a relatively high possibility. Thus, further analysis and personalized medicines can be successfully launched.

The computational complexity of analyzing SNP interactions has been confirmed as a NP hard problem [3, 4]. As such, a global optimal solution is difficult to be located in a combination space, especially when the number of SNPs is large. Numerous methods were proposed to balance the quality of solutions and calculation costs, and many approaches tried to find near optimal solutions in such a huge combination space. For example, intelligent algorithms can be suitable; Chang et al. [2] used a particle swarm optimization algorithm to search for barcodes with a high risk. Yang et al. [3] improved a genetic algorithm by using a selective strategy to optimize the maximum difference. A heuristic search strategy can also be applied to analyze SNP interactions. For instance, Chuang et al. [4] designed a branch-and-bound algorithm to narrow down the combination space of SNP interactions.

Although these approaches have some merits, some issues should be addressed in further. For instance, some approaches only focus on the difference in barcodes frequencies between cases and controls. In our study, the consistency of barcodes is simultaneously considered to identify the risky barcode related to these cases. In another instance, some methods intend to search for a specific barcode, which is globally optimal [5]. However, these cases can be precisely divided into several subtypes on the basis of the trait heterogeneity of a complex disease [6]. It means that there may be several different risky barcodes related to different subtypes of complex diseases. In our study, an improved ant colony algorithm is proposed to address these problems and to generate multiple solutions; in each iteration, optimum solutions are saved as candidate barcodes attributed to a specific subtype of complex diseases. More importantly, traditional methods make the preference that odds ratio (OR) or risk ratio (RR) produces plausible barcodes, which yield a high OR but exhibit a low frequency in the case group or a high frequency in the control group.

Our study aims to address all these three issues in the process of generating risky barcodes. To search feasible solutions in the huge combination space of SNP interactions, an ant colony optimization (ACO) is applied to identify multiple risky barcodes with criteria maximum discrepancy and maximum consistency (MDMC). Note that we provide a new way to determine the length of risky barcodes according to the frequency of barcode. This is because from statistical point these barcodes should meet a reasonable frequency in cases or controls.

## Methods

In Eq. (1), a matrix *M* contains *m* samples, and each sample includes *n* SNPs and a label which indicates the status of sample (normal or effected). Each *x*_*i*,*j*_ entry denotes genotype of *j*th SNP on the *i*th sample; in this study, *x*_*i*,*j*_ could be 0 (major allele homozygous), 1 (heterozygotes) or 2 (minor allele homozygous). For a case–control study, *l* could be 0 (control) or 1 (case).

1 $$ M=\left[\overset{SNPs}{\overbrace{\begin{array}{cccc}\hfill {x}_{1,1}\hfill & \hfill {x}_{1,2}\hfill & \hfill \cdots \hfill & \hfill {x}_{1, n}\hfill \\ {}\hfill {x}_{2,1}\hfill & \hfill {x}_{2,2}\hfill & \hfill \cdots \hfill & \hfill {x}_{2, n}\hfill \\ {}\hfill \vdots \hfill & \hfill \vdots \hfill & \hfill \hfill & \hfill \vdots \hfill \\ {}\hfill {x}_{m,1}\hfill & \hfill {x}_{m,2}\hfill & \hfill \cdots \hfill & \hfill {x}_{m, n}\hfill \end{array}}}\overset{Label}{\overbrace{\begin{array}{c}\hfill {l}_1\hfill \\ {}\hfill {l}_2\hfill \\ {}\hfill \vdots \hfill \\ {}\hfill {l}_m\hfill \end{array}}}\right] $$

In this work, a MDMC measure is designed to determine a risky barcode which is highly associated with labels of samples. ACO algorithm is applied to generate these risky barcodes. Thus, our method can be denoted by MDMC-ACO.

### MDMC

Let **X** be the random variable of barcode and **X** takes the values of the barcode **x**_*i*_ = {*x*_*i*1_, … *x*_*ij*_, … *x*_*in*_}, where *xij* is the genotype on *i*-th barcode at the SNP *j*, *i* ∈ {1, …, m}, *j* ∈ {1, …, n}. Then, let *pcase*(**x** *i*) be the frequency of **x** *i* on cases and let *pcontrol*(**x** *i*) be the frequency of **x** *i* on controls.

*Maximum discrepancy(MD)*: Intuitively, a barcode, which causes a complex disease, should appear frequently in cases but rarely in controls. The discrepancy of frequencies should also be as large as possible. MD is described in Eq. (2).

2 $$ \max\ \mathrm{D}\left(\mathbf{x}\mathrm{i}\right),\  D= pcase\left(\mathbf{x}\mathrm{i}\right)- pcontrol\left(\mathbf{x}\mathrm{i}\right) $$

When D is negative, **x**i is a weak solution. A more risky barcode should yield a larger value. SNPs selected according to MD could be redundant or noisy. For example, the discrepancy could be constant when a new SNP is added to a barcode. Therefore, the following maximum consistency condition can be used to restrain noisy SNPs.

*(Maximum consistency)MC*: If no other factors result in these cases except genes, these cases should be attributed to several risky barcodes. At the same time, these barcodes should never appear in controls. Although other unknown factors undermine this ideal situation, MC with cases should be maintained to exclude noise.

3 $$ \max\ \mathrm{C}\left(\mathbf{X}\right),\ \mathrm{C}=\frac{1}{{\displaystyle \sum_{i=1}^m p\left(\mathbf{x} i\right) log\frac{p\left(\mathbf{x} i\right)}{{\displaystyle {\prod}_{j=1}^n p j(xij)}}}} $$

Equation (3) describes relative entropy, which is a fundamental measure in information theory [7, 8]. If *p*(**x** *i*) remains unchanged when a new SNP is selected, ∏_*j* = 1_^*n*^ *pj*(*xij*) decreases. Consequently, *C* decreases. It means that an unreasonable growth of the number of causing SNPs is restrained.

### ACO algorithm

As the number of SNPs increases, the combination space of epistasis grows rapidly, which results in that a global optimal solution cannot be searched in such a huge space. Therefore, ACO algorithm is proposed to heuristically search for near-optimal solutions.

The ACO algorithm, which is a classic swarm intelligence method, solves computational tasks by using a probabilistic technique [9]. ACO was initially proposed to solve an optimal path for the traveling salesman problem. ACO has been widely applied to various research areas. ACO simulates the behaviors of natural ants as they search for foods; some of these behaviors include laying down pheromone and so on. In the following subsections, the design of ACO is detailed.

#### Population encoding and initialization

In accordance with the risky barcode searching problem, each individual *I* of ACO, namely, barcode, is designed in a format on the basis of the selected SNPs and genotypes on these SNPs. The encoding scheme is expressed as follows:

$$ I=\left\{\left( SNP, Genotype\right)1,\dots, \left( SNP, Genotype\right) k\right\} $$

where each component of *I* contains the ID of SNP and the genotype of the selected SNP. *k* is the number of SNPs in a barcode.

In the traditional ACO, ants can be randomly placed on SNPs. In this study, each artificial ant is distributed in a fixed distance because of the linkage disequilibrium (LD) between adjacent SNPs. For example, suppose that there are *w* ants and *n* SNPs. Then, all of these SNPs in a chromosome are divided into *w* segments. During the ACO initialization, the *i*-th ant can only be placed in the *i*-th segment, but the ant can randomly select a SNP in each segment. This strategy can improve global optimality.

#### Objective function

The processes of SNP selection and barcode generation are determined by an objective function. In this study, each solution generated by ACO will be evaluated by MDMC, where MD and MC are combined in Eq. (4).

4 $$ \max\ \mathrm{F}\left( D, C\right),\ \mathrm{F}=\mathrm{D}\times \mathrm{C} $$

Obviously, Eq. (4) is the simplest combination formula of MD and MC to maximize MDMC. Intuitively, a risky barcode which is highly correlated to the progress of complex diseases will yields a higher *F*.

#### SNP selection and pheromone update

A key communication medium of ACO that directs an ant’s decision-making ability is pheromone. If a SNP is selected by many ants, the pheromone on the SNP is rich. Therefore, the possibility of other ants selecting it to formulate a risky barcode is high. The ant decision of SNP selection is defined as follows:

5 $$ {\mathrm{p}}_{\mathrm{i}}^{\mathrm{k}}\left(\mathrm{t}\right)=\left\{\begin{array}{l}\frac{{\left[\uptau \mathrm{i}\right]}^{\upalpha}\bullet {\left[\upeta \mathrm{i}\right]}^{\upbeta}}{{\displaystyle \sum_{i\in R}{\left[\uptau \mathrm{i}\right]}^{\upalpha}\bullet {\left[\upeta \mathrm{i}\right]}^{\upbeta}}}\ \mathrm{i}\in \mathrm{R}\\ {}0\kern0.75em \mathrm{Otherwise}\end{array}\right. $$

R is the set of unresolved SNPs, and α and β are two important weights of pheromone trail and heuristic value, respectively. A heuristic value η, which can accelerate convergence, is essential for analysis of large-scale dataset. When previous studies have found some risky SNPs which are pathogenic to a specific complex disease through clinical validation, we can set a high η for these risky SNPs to ensure that they will be selected with relatively high probability. However, we suppose that risky barcodes are identified from few candidate SNPs in this study. Heuristic information is unnecessary; thus, β is 0.

Once all the ants have searched for their solutions in each iteration, laying down pheromone and evaporation in all of the SNPs are triggered. In accordance with the objective function, the pheromone update of τ is defined as Eq. (6).

6 $$ \uptau \mathrm{i}\left(\mathrm{t}\right)=\left(1\hbox{-} \uprho \right)\uptau \mathrm{i}\left(\mathrm{t}\hbox{-} 1\right)+\varDelta \uptau \mathrm{i}\left(\mathrm{t}\right) $$

where ρ is the pheromone evaporation factor and ρ ∈ (0, 1). *Δ*τi(t) is expressed as Eq. (7).

7 $$ \varDelta {\tau}^k\mathrm{i}\left(\mathrm{t}\right)=\left\{\begin{array}{l}\mathrm{Fk}\ \mathrm{i}\in {\mathrm{T}}^{\mathrm{k}}\left(\mathrm{t}\right)\\ {}0\kern0.75em \mathrm{Otherwise}\end{array}\right. $$

where T^k^(t) is the set of SNP selected at the *t-*th iteration. Eq. (7) denotes that the *k-*th ant deposits a quantity of pheromone *Δ*τi(t) on the SNPs involved in a potential barcode.

#### Determining the length of barcode

A pathogenic barcode could not be too frequent in controls and also could not be too infrequent in cases from a statistical view. According to this property, some noisy barcodes can be excluded, and oversize barcodes will be inhibited. Therefore, we introduce a noisy pruning strategy to determine the length of barcode in ACO. The length of a risky barcode is bound to a reasonable region neither too long nor too short.

During the process of barcode generation, we find that if a short barcode is infrequent, its superset is also infrequent. It means that if an ant generates a solution which is too infrequent in cases, there is no need to continue. Consequently, instead of expanding the size of barcodes, the ant then returns a short barcodes.

### Pseudo-code of MDMC-ACO

**Fig. 1:**
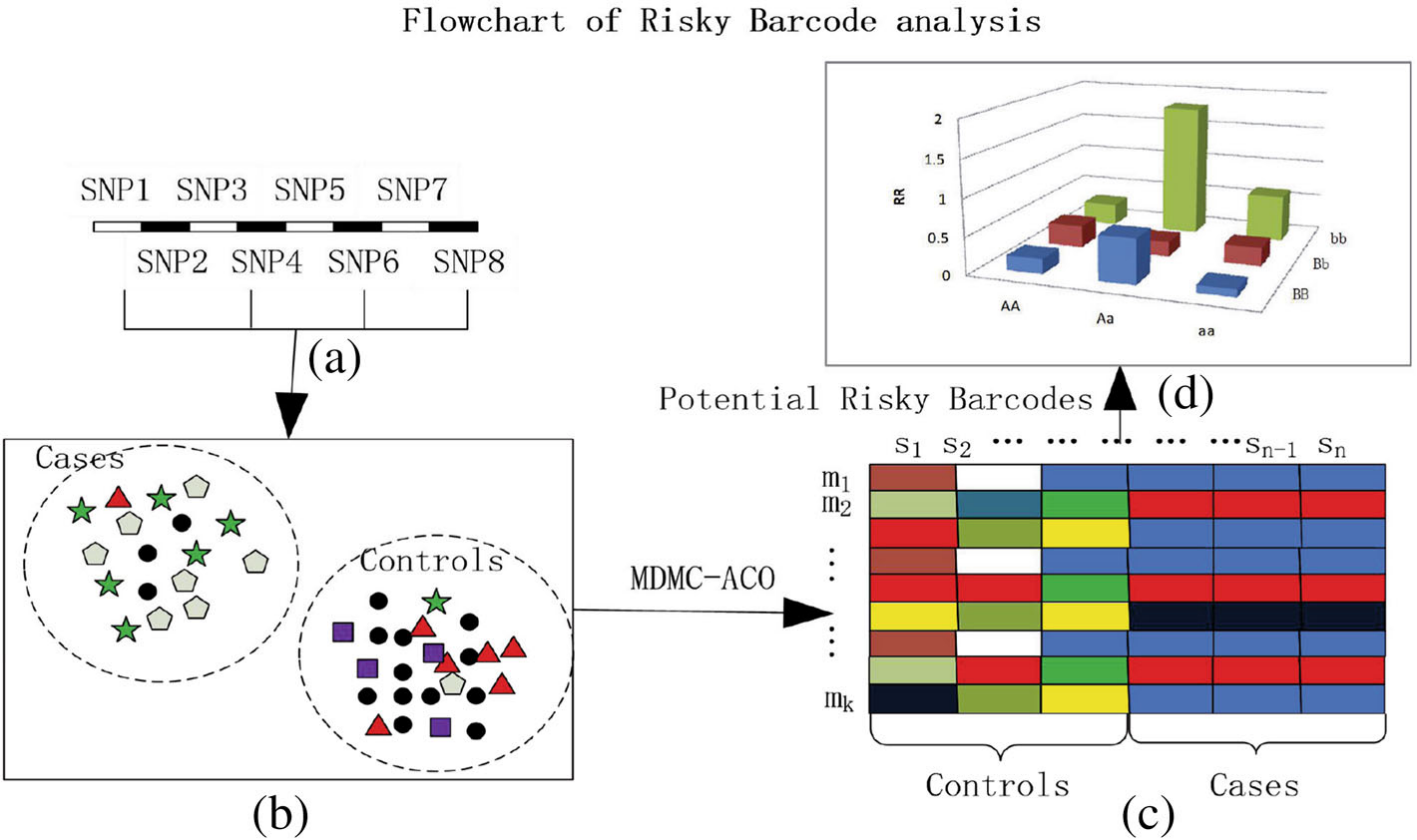
Searching multiple potential barcodes by MDMC-ACO. For candidate SNPs, MDMC-ACO considers both epistasis and trait heterogeneity in case–control study. Multiple barcodes generated by MDMC-ACO may related to different subtypes of complex diseases.


The pseudo-code is listed below to describe MDMC-ACO and the outline of searching for potential risky barcodes is depicted in Fig. 1.

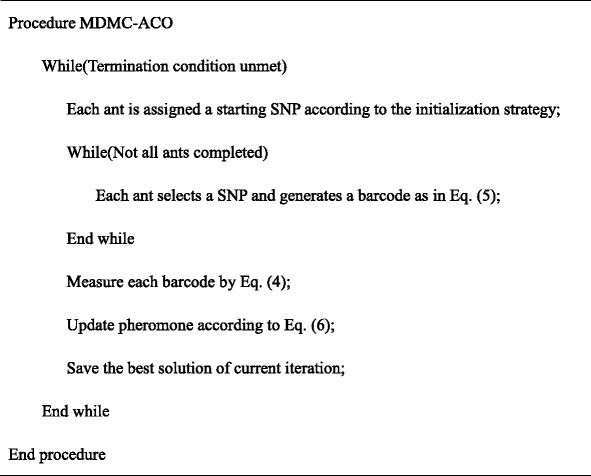


The first loop ends when a specific amount of iterations is reached or a fixed number of risky barcodes is satisfied.

## Datasets and performance measurement

### Dataset of breast cancer

The predictive barcodes related to a specific complex disease is identified through the following steps: (1) use GWAS to identify risky SNPs by a case–control study; (2) run a permutation test for these SNPs; (3) deeply sequence the mutation around candidate SNPs; (4) carry out a candidate association study to determine SNPs in epistasis; and (5) search for pathogenic barcodes.

In this study, risky barcodes of breast cancer are searched from a dataset which contains a candidate set of SNPs and 10,000 samples (5000 cases and 5000 controls). Twenty-three candidate SNPs which are separately distributed in six genes, namely, COMT, CYP19A1, ESR1, PGR, SHBG, and STS are involved in the progress of breast cancer. We use the same number to represent the ID of each SNP, which is consistent with previous study [3]. These genes involved in steroid hormone metabolism and signaling are verified in [10–12]. Then, risky barcodes should also be analyzed to determine the harmful genotype of these genes in breast cancer.

### Performance measurement

We apply two common measures, namely, OR and RR, to evaluate the performance of risky barcodes. These measures are widely used in epidemiological and case–control studies.

8 $$ OR=\frac{TP\times TN}{FN\times FP} $$

9 $$ R R=\frac{TP\times \left( TN+ FP\right)}{FP\times \left( TP+ FN\right)} $$

A predictive barcode can be used for early diagnosis or risk estimation. If a predictive barcode is considered as pathogenic, then an individual carrying the barcode would be diagnosed as positive. In Eqs. (8) and (9), TP denotes the proportion of cases carrying the predictive barcode, FP represents the proportion of controls holding the predictive barcode, and TN is the proportion of controls that do not carry the barcode, and FN is the proportion of cases that do not carry the barcodes.

## Results

IGA is a method that applies an efficient strategy to improve genetic algorithms for generating potential barcode [3]. However, IGA still faces several issues. For instance, IGA does not consider trait heterogeneity. The only optimal solution of IGA ignores the multiple subtypes of complex diseases. Furthermore, IGA only uses the maximum difference as a fitness function, so that it cannot exclude noisy barcodes which exhibit high maximum difference with unreasonable frequency. In addition to these drawbacks, a long risky barcode could be generated, while a short barcode more likely occurs than a long barcode. In the following subsections, comprehensive results are described.

### Comparison of MDMC-ACO and IGA on OR and RR

**Fig. 2:**
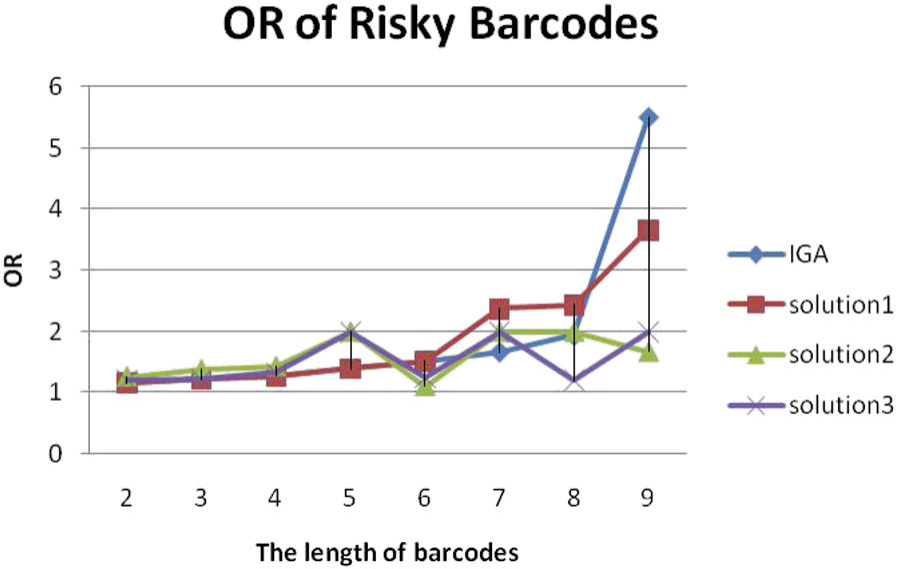
OR values of various barcodes with different lengths. IGA denotes OR value of the most risk barcode generated by IGA method. The *solution1*, *solution2* and *solution3* are three different risk barcodes generated by MDMC-ACO. The unit of the length of barcodes is one SNP. It means that when the length is 6, there are 6 SNPs in a barcode.

**Fig. 3:**
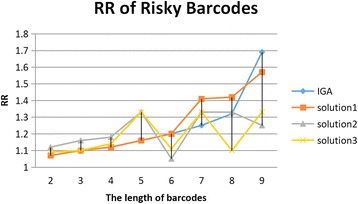
RR values of various barcodes with different lengths. IGA represents RR value of the most risk barcode generated by IGA method. The *solution1*, *solution2* and *solution3* are three different risk barcodes which may lead to different subtypes of complex diseases. The unit of the length is one SNP.

Because our method MDMC-ACO can generate multiple barcodes, we choose three different risky barcodes denoted as *solution1*, *solution2*, and *solution3*, respectively, and compare with those of IGA. The maximum length of these barcodes is 9 because our method restricts the frequency of barcodes. The comparison results of OR and RR are depicted in Figs. 2 and 3.

For *solution1*, these results of OR and RR are equal to IGA when the length of barcodes is shorter than 7. As the length increases, *solution1* is generally better than IGA. By contrast, *solution3* sustains its advantages as the length increases except when the length is equal to 6 and 9. The performance of *solution3* is also different from IGA and *solution1*. For *solution2*, it is more risky than other solutions at the beginning. However, the pattern of *solution2* is disrupted when other noisy SNPs are selected. Although the performance of *solution2* declines, *solution2* is also risky. It means that these barcodes are at different level of risk. These risky barcodea could lead to different subtypes of complex diseases, which indicates that our method can identify risky barcodes correlated with multiple subtypes. The details of these solutions of our method are discussed in the following sections.

### Multiple risky barcode details

Multiple subtype patterns may hidden in cases because of trait heterogeneity. In this study, ACO is designed to obtain multiple risky barcodes correlated with subtypes of breast cancer. The details of these three barcodes are listed in Tables 1, 2 and 3. The maximum length of risky barcodes is set as 6 since a shorter barcode is statistically easier to form from statistical view.

**Table 1:** Details of *solution1*

Epistatic SNPs	Genotype Barcode	Cancer percentage (%)	RR (95% CI)	OR (95% CI)
4-17	2-1	52.75	1.07 (1.0254, 1.1217)	1.15 (1.0506, 1.2662)
4-17-18	2-1-1	54.19	1.1 (1.0355, 1.1655)	1.21 (1.0657, 1.3687)
4-17-18-22	2-1-1-2	55.60	1.12 (1.0347, 1.2102)	1.27 (1.0644, 1.5107)
4-11-17-18-22	2-2-1-1-2	57.98	1.16 (1.0473, 1.2946)	1.39 (1.0826, 1.788)
4-11-16-17-18-22	2-2-2-1-1-2	60.00	1.2 (1.0347, 1.3982)	1.51 (1.0356, 2.193)
4-11-12-16-17-18-22	2-2-1-2-1-1-2	70.27	1.41 (1.1403, 1.7373)	2.37 (1.1702, 4.8032)

**Table 2:** Details of *solution2*

Epistatic SNPs	Genotype Barcode	Cancer percentage (%)	RR	OR
2-4	0-0	55.69	1.12 (0.9974, 1.2509)	1.26 (0.9799, 1.6308)
2-4-6	0-0-1	57.97	1.16 (0.9485, 1.4203)	1.38 (0.8557, 2.2332)
2-4-6-7	1-0-1-0	58.82	1.18 (0.7903, 1.7525)	1.43 (0.5437, 3.7583)
2-4-6-7-20	0-0-0-0-0	66.67	1.33 (0.5989, 2.9689)	2 (0.1813, 22.0689)
2-4-6-7-17-20	0-0-2-2-1-1	52.38	1.05 (0.6965, 1.5760)	1.1 (0.4668, 2.5929)
2-4-6-7-12-17-20	2-2-1-1-0-1-1	66.67	1.33 (0.5989, 2.9689)	2 (0.1813, 22.0689)

**Table 3:** Details of *solution3*

Epistatic SNPs	Genotype Barcode	Cancer percentage (%)	RR	OR
4-8	0-1	54.31	1.09 (0.9821, 1.2081)	1.2 (0.9537, 1.4985)
4-6-8	0-1-1	54.9	1.1 (0.9209, 1.3120)	1.22 (0.8243, 1.8053)
4-6-8-19	0-2-1-0	57.14	1.14 (0.6016, 2.1717)	1.33 (0.2983, 5.9618)
4-6-8-19-20	2-2-2-0-0	66.67	1.33 (0.5989, 2.9689)	2 (0.1813, 22.0689)
4-6-8-13-19-20	0-0-1-0-1-2	55.56	1.11 (0.6193, 1.9940)	1.25 (0.3355, 4.6587)
4-6-8-13-18-19-20	0-0-2-2-1-1-2	66.67	1.33 (0.5989, 2.9689)	2 (0.1813, 22.0689)

When OR and RR of the barcodes are bigger than 1, the barcodes can be considered risky. When there are several different barcodes regarded as risk factors, the barcodes may represent different patterns correlated with different subtypes of breast cancer. Once the barcodes have been ranked according to the degree of risk, researchers can select the top barcodes for further clinical analysis.

From the results of 95% CI on OR and RR in Tables 1, 2 and 3, although these barcodes meet the criteria MD and MC and satisfy the requirement of OR and RR, not all of them meet a satisfied significant level. It is because that we have not set limitation directly on statistical significance during searching solutions. To further analyze statistical significance, we run our method three times and calculate the *p*-value of several potential barcodes in these three solutions as shown in Table 4.

**Table 4:** Statistical analysis of risky barcodes

Epistatic SNPs	Genotype Barcode	*p*-value
4-17	2-1	0.003
4-17-18	2-1-1	0.003
4-17-18-22	2-1-1-2	0.008
4-11-17-18-22	2-2-1-1-2	0.012
4-11-16-17-18-22	2-2-2-1-1-2	0.031
4-11-12-16-17-18-22	2-2-1-2-1-1-2	0.013
2-4	0-0	0.071
2-4-6	0-0-1	0.184
4-8	0-1	0.121
4-6-8	0-1-1	0.319

Barcodes listed in Table 4 are the most frequent solutions saved by MDMC-ACO in different run configurations. The barcodes located in the first six rows in Table 4 are statistically significant (*p*-value < 0.05). In addition to statistical significance, we will give further analysis on pathogenic SNP and frequency of barcodes in next subsections.

### The most common pathogenic SNP

Although the subtypes of complex diseases may exhibit different properties, these subtypes likely display a common feature to some extent. Therefore, some common SNPs may be involved in various risky barcodes. In this study, these potential barcodes obtained by MDMC-ACO are further analyzed. We find that among these barcodes, SNP 4 (rs3020314) on the ESR1 whcih is the most common SNP appears in all risk barcodes.

MDMC-ACO has generated numerous intermediate results during the process of optimization. We analyze these intermediate results and find that once a barcode carries rs3020314, the value of OR and RR would be increase. In previous studies [13–15], ESR1 encodes an estrogen receptor, a ligand-activated transcription factor composed of several domains essential for hormone binding, DNA binding, and transcription activation. ESR1 also participates in pathological processes, such as breast cancer, endometrial cancer, and osteoporosis. These biological functions of ESR1 verify the statistical results of our method.

### Determination of the length of barcodes

The length of barcodes should be restricted due to two reasons : 1) For example, the OR and RR of *solution1* increase as the length of barcodes grows. However, the optimum combination remains unknown. 2) If a short barcode is risky and pathogenic, then the short barcode more likely forms than a long barcode. However, a very short barcode may lead to information loss and false positive results may be obtained. Therefore, it is important to determine the length of potential barcodes.

To address these issues, we apply a further restriction on the frequency of these barcodes. Suppose that a complex disease only contains one kind of subtype and no other environmental factors influence the subtype. In a ideal case, patients likely carry one type of barcodes, and this barcode never exists in normal situations. However, other genetic or environmental factors can disrupt the ideal situation. Thus, the pathogenic pattern can be more complex in case–control studies: 1) multiple barcodes may be found in cases; 2) risky barcodes exist in controls.

Although the ideal situation is disturbed and complex situations possibly occur, it cannot be too extreme. In other words, risky barcodes cannot be too infrequent in cases and the barcode cannot also be too common in controls. In this study, a threshold of frequency is set as 1%. This finding indicates that the frequency of each risky barcode should satisfy the following condition: the frequency should be <1% in controls and the frequency should be >1% in cases. A total of 5000 cases and 5000 controls are considered in our study. Therefore, the number of patients carrying the barcode should be >50 and the number of normal people carrying the barcode should be <50. Two risky barcodes correlated to breast cancer survive because of this restriction, and their lengths are 6 and 3, respectively. One barcode is composed of 4-11-17-18-21-22 (rs3020314-rs9340799-rs660149-rs11571171-rs272428-rs858524) whose frequencies in cases and controls are 70 and 47, respectively. Another barcode is composed of 4-6-8(rs3020314-rs1543404-rs2747652) whose frequencies in cases and controls are 56 and 46, respectively.

## Discussion

In this study, an intelligent algorithm is applied to optimize constraints MD and MC for identifying pathogenic barcodes. However, our approach cannot guarantee an optimal and stable solution for this kind of problem since ant colony algorithm is involved in probabilistic techniques. Despite this limitation, our method commits several merits as forementioned.

The results of our method are detailed in different aspects. IGA is compared with MDMC-ACO to validate the effectiveness of our method. These results reveal that our method can effectively determine pathogenic risky barcodes. Another merit of our method is that MDMC-ACO can generate multiple solutions which are related to different subtypes of complex diseases because of trait heterogeneity. Some of these barcodes are dominant since their OR and RR are significantly >1. A common ground is likely found among these different subtypes of complex diseases. It means that there are several common SNPs referred to different subtypes. Therefore, these common pathogenic SNPs need be further understood by gene function analysis.

In addition, considering the restriction of the barcode frequency, we can determine the optimum length of barcodes and exclude noisy barcodes. Multiple barcodes are regarded as risk factors of complex disease. Noise may exist in these solutions. Filter strategy can be applied. For example, these barcodes can be ranked on the basis of OR and RR. These top barcodes are dominant in cases.

The barcode rs3020314-rs1543404-rs2747652 whose OR and RR are 1.1 and 1.2 contains three SNPs and all of them exist in ESR1. Another barcode rs3020314-rs9340799-rs660149-rs11571171-rs272428-rs858524 contains six SNPs located in ESR1, PGR, and SHBG, separately; the OR and RR of this barcode are 1.2 and 1.5, respectively. All of these genes are correlated with breast cancer [16–19].

Another challenging issue of epistasis analysis is computational complexity. In the pseudo-code of MDMC-ACO, the complexity is *Θ*(*I* × *A* × *L* × *m* × *n*), where *I* is the number of the maximum iterations of ACO, and *A* is the amount of artificial ants, and *L* is the average length of barcodes, and *m* is the number of samples and *n* is the number of SNP.

## Conclusions

In this study, MD and MC criteria are combined with ACO to identify risky barcodes related to breast cancer. MDMC-ACO has addressed several important issues. The results show that our method can be used for the epistasis analysis of complex diseases. Although MDMC-ACO can identify several risky barcodes, these barcodes should be further validated in clinical studies.

Although our method provides several advantages, further studies should be performed 1) to use other complex diseases to evaluate MDMC; 2) to design an algorithm appropriate for GWAS and candidate studies; and 3) to develop a software with friendly GUI.

## Abbreviations

ACO: Ant colony optimization
GWAS: Genome-wide association studies
MDMC: Maximum discrepancy and maximum consistency
OR: Odds ratio
RR: Risk ratio
SNPs: Single nucleotide polymorphisms
